# Rapid fabrication of biomimetic PLGA microsphere incorporated with natural porcine dermal aECM for bone regeneration

**DOI:** 10.1093/rb/rbae099

**Published:** 2024-08-26

**Authors:** Xiaosong Zhou, Min Guo, Zongliang Wang, Yu Wang, Peibiao Zhang

**Affiliations:** Key Laboratory of Polymer Ecomaterials, Changchun Institute of Applied Chemistry, Chinese Academy of Sciences, Changchun 130022, P. R. China; School of Applied Chemistry and Engineering, University of Science and Technology of China, Hefei, Anhui 230026, P. R. China; Key Laboratory of Polymer Ecomaterials, Changchun Institute of Applied Chemistry, Chinese Academy of Sciences, Changchun 130022, P. R. China; Key Laboratory of Polymer Ecomaterials, Changchun Institute of Applied Chemistry, Chinese Academy of Sciences, Changchun 130022, P. R. China; Key Laboratory of Polymer Ecomaterials, Changchun Institute of Applied Chemistry, Chinese Academy of Sciences, Changchun 130022, P. R. China; Key Laboratory of Polymer Ecomaterials, Changchun Institute of Applied Chemistry, Chinese Academy of Sciences, Changchun 130022, P. R. China; School of Applied Chemistry and Engineering, University of Science and Technology of China, Hefei, Anhui 230026, P. R. China

**Keywords:** extracellular matrix, electrohydrodynamic spraying, biomimetic microsphere, bone tissue engineering

## Abstract

Bioactive microspheres coated with acellular extracellular matrix (aECM) have received extensive attention in bone tissue engineering. In this work, biomimetic microspheres with different aECM ratios, uniform size and controllable size were prepared easily by blending natural porcine dermal aECM and poly (lactic-co-glycolic acid) (PLGA) using electrohydrodynamic spraying and solidification actuated by solvent extraction method. In this work, the appropriate polymer concentration and preparation voltage were investigated, and the surface morphology of the microspheres was observed by scanning electron microscope. Sirius red was used to visualize aECM exposure on the surface of the microspheres. The *in vitro* and *in vivo* experiments were carried out to evaluate the bioactivity and osteogenic properties of the microspheres. The results showed that the morphology and size of PLGA microspheres had little influence on the aECM blending. *In vitro* experiments showed that the higher the content of aECM, the better the cell adhesion performance. *In vivo*, rat calvarial defect models were observed and characterized at 4 and 8 weeks postoperatively, and the values of BV/TV of 50aECM/PLGA were 47.57 ± 1.14% and 72.92 ± 2.19%, respectively. The results showed that the skull healing effect was better in aECM-containing microspheres. In conclusion, aECM/PLGA composite microspheres can increase cell adhesion performance through the addition of aECM. Moreover, *in vivo* experiments have proved that aECM/PLGA microspheres are beneficial to bone repair, which means the aECM/PLGA microspheres are a promising bone tissue engineering material.

## Graphical abstract

**Figure:**
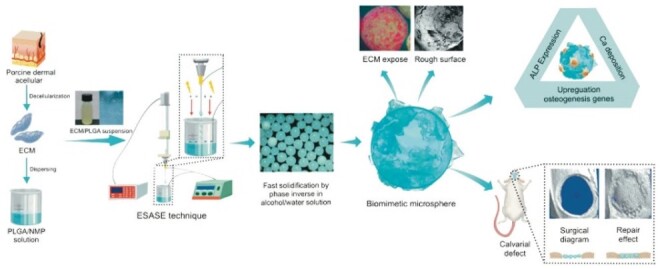

## Introduction

Large bone defects caused by disease or trauma have become a major clinical challenge in orthopedics because of their difficulty in repairing [1, 2]. With more than 2 million bone graft procedures performed annually, the global market size for bone scaffolds and substitutes will reach US $4.29 billion by 2026 [3, 4]. The gold standard of clinical bone defect repair is autogenous bone graft, but the limited source of donors, secondary injury and infection risk restrict the application of autologous bone transplantation [5, 6]. Therefore, artificial bone substitutes based on various biological materials have attracted increasing attention [7–9]. Besides, owing to the irregular shape of the defect features, the large bone defect is difficult to repair [10, 11]. Therefore, there is an urgent need to design and develop new bone substitutes for experimental studies and clinical applications in orthopedics. The characteristics of the ideal bone substitute should be both osteoconduction and osteoinductivity [12–14]. They should also be able to support cell adhesion, growth and extracellular matrix (ECM) formation [15].

Recently, as a new type of spherical scaffold material, microspheres with biological functions have received extensive attention in bone tissue engineering [16, 17]. Compared with other types of scaffolds, microspheres are characterized by providing a high specific surface area for cell growth and maintaining a differentiated cell phenotype [18, 19]. They can serve as the basic building blocks for constructing scaffolds, and the micrometer-sized gaps between them can allow oxygen and nutrients to enter while supporting cell growth [17]. At present, the most commonly used methods for preparing microspheres include emulsion methods [20, 21], spray-drying methods [22, 23], microfluidic methods [24, 25] and electrohydrodynamic (EHD) spraying techniques [26, 27]. The microspheres prepared by the emulsion and spray-drying method are inhomogeneous, and further screening is needed. In addition, the whole preparation process takes a long time. Although the microfluidic method has good uniformity, the device for microfluidic method is complex. Besides, this method is cumbersome to operate, costly and has poor preparation efficiency. EHD spraying techniques work on the principle of high-voltage electrostatic field between two charged electrodes generating a jet to fabricate drops [28]. In the case of water-soluble solvents, injection of a polymer solution into the receiver resulted in a rapid diffusion of N-methyl pyrrolidone (NMP) into the received solution. The solution was immediately quenched and triggered the solidification of the polymer [29]. This method is simple to operate and enables continuous preparation of microspheres with uniform size and customizable size in a controlled manner by adjusting various parameters, including media properties (e.g. concentration/viscosity, conductivity) and applied voltage [30–32].

Poly (lactic-co-glycolic acid) (PLGA) is a co-polymer which is a promising biodegradable biomaterial which is approved by FDA [33, 34]. The polymer displays good elastic mechanical properties and the degradation rate can be regulated [35]. However, the biological inertness seriously limits its biological applications [36]. To overcome these obstacles, many bioactive inorganic or organic materials have been synthesized or modified onto PLGA, such as hydroxyapatite [37–39], growth factor [40–42], ECM [15, 43, 44] and so on. Among them, ECM is rich in collagen and serves as a microenvironment for cell growth and thereby regulate tissue regeneration on by modulating cell functions and now widely used in tissue engineering and biomaterials [45]. However, the mechanical properties of ECM are poor, and composite polymer materials are usually needed to improve the mechanical properties [46]. Zhou *et al.* [15] used decellularization technology to generate derived ECM on the surface of PLGA microspheres to achieve the purpose of increasing the bioactivity of the surface of microspheres. In this way, the microstructure of ECM can be well preserved to maximize the role of ECM. Pathak *et al.* [47] chemically grafted ECM onto PLGA by Schiff base reaction to increase its surface bioactivity. This method allows the aECM to be firmly attached to the PLGA surface with controllable cut-off, but the chemical method is complex to operate and poses safety risks. Kim *et al.* [48] directly blended collagen and PLGA to increase the anti-inflammatory and osteogenic properties of the material. This method can combine collagen and PLGA simply and efficiently and exert certain anti-inflammatory and osteogenic functions, but it is a single component of ECM without the microstructure of ECM. Therefore, the utilization of microstructurally intact ECM and the development of simpler preparation methods are very necessary. In this study, aECM derived from porcine skin without immunogenicity was used. Besides, in this work, aECM and PLGA were composite by simple blending using EHD spraying and solidification actuated by solvent extraction (ESASE) technique, as a short-time consuming and costly procedure.

In this study, aECM/PLGA microspheres were fabricated rapidly by ESASE technique. In this research, the voltage and concentration of PLGA solution was studied. Subsequently, biomimetic aECM/PLGA microspheres with different aECM contents were prepared and the surface morphology and aECM distribution on the surface of the microspheres were observed by SEM and Sirius red staining. The aECM/PLGA microspheres can promote the adhesion and osteogenic differentiation of MC3T3-E1 by *in vitro* evaluation. The rat calvarial defect experiment revealed that the bioactive microspheres can promote bone defects recovery. Through the detection of microspheres and *in vitro* and *in vivo* experiments, a fast and convenient method for the preparation of bioactive microspheres has been developed and shows a promising application in bone tissue engineering.

## Experimental

### Materials

L-lactide (LA) and glycolide (GA) were purchased from Purac (Netherlands), Holland. Stannous octoate (Sn(Oct)_2_) was obtained from Sigma. Acellular ECM (aECM) mADM^®^ purchased from Unitrump Bio (Jiangsu). All chemicals were of analytical grade or higher. Fetal bovine serum (FBS) and Dulbecco’s modified Eagle’s medium (DMEM) were purchased from Gibco (New York, USA). Penicillin and streptomycin were obtained from Solarbio (China). Cell Counting Kit-8 (CCK-8) was purchased from Dojindo Molecular Technologies, Inc. NMP, calcein-AM and propidium iodide (PI) were purchased from Aladdin (China). Polyformaldehyde (PFA) was purchased from Macklin (China). Alkaline phosphatase (ALP) assay kits were obtained from Beyotime (China) and Alizarin Red (AR) and cetylpyridinium chloride (CPC) were obtained from Sigma (USA). PrimeScript RT Reagent Kit with the gDNA Eraser RR047A and SYBR Premix Ex Taq RR420A were purchased from TaKaRa (Japan). Mouse pre-osteoblast MC3T3-E1 cells were purchased from Institute of Biochemistry and Cell Biology, Shanghai Institutes for Biological Sciences.

### Fabrication of aECM/PLGA microspheres

PLGA (LA/GA = 80:20) with the viscosity’s average molecular weight of 92 460, was synthesized by the ring opening copolymerization of the LA and GA in the presence of stannous octoate (Sn(Oct)_2_) as a catalyst and isopropanol as the initiator [2]. Extraction of type I collagen was the later procedure [49, 50], the rat tail was used as raw material, the tendon in the tail was stripped, dissolved in acetic acid solution with a volume fraction of 0.5 mol/l, stirred at 4°C for 3 days, the obtained solution was filtered through the filter to obtain collagen solution, then salting out with 20% sodium chloride, and the precipitate was collected by centrifugation. The obtained precipitate was dialyzed for one week and lyophilized to obtain the product. The resulting collagen was crushed by a crusher for subsequent experiments.

aECM/PLGA microspheres were fabricated utilizing an ESASE technique. The preparation device was made in our lab as before [1, 30]. Briefly, the microsphere preparation device comprised five parts: the supporting frame, an extruder, a high voltage electrostatic (HVE) generator, a stepper motor controller and collecting device which contains collecting solution. A standard plastic syringe (2 ml) loaded with stainless steel dispensing needle (19 gauge) is fixed in the supporting frame and controlled by the extruder and controlled and the stepper motor controller. The HVE generator applied high voltage between the dispensing needle and receiving device. The positive electrode was connected to the dispensing needle and the negative electrode was connected to the collecting device. The extruder pushed the solution to the tip of the nozzle. Under the influence of electrostatic force and gravity, the drips drop into the collecting device. The solvent quickly got into the collecting solution and the PLGA crystallized rapidly and solidified. In this way, biomimetic PLGA microspheres were fabricated rapidly.

The PLGA was dissolved in NMP with a range of concentrations (5%, 7%, 9%w/v) and the viscosity of each solution was tested by NDJ-8S smart viscometer (NiRun, Shanghai). Then the solutions were electro jetted into collecting device which contains ethanol aqueous solution with concentration of 50% at room temperature, and the distance between the nozzle and collecting device is 5 cm. To study the effect of voltage on the microsphere properties, different voltages between the stainless steel nozzles and ethanol solution were applied (4.5, 5.0, 5.5, 6.0, 6.5, 7.0 and 7.5 kV). As shown in Figure 3B, the aECM powder was evenly dispersed in NMP solution of PLGA with homogenizer to obtain homogeneous systems with the concentration of 1%, 5%, 10%, 25%, 50% (compared to PLGA) and the prepared microspheres were recorded as 1aECM/PLGA, 5aECM/PLGA, 10aECM/PLGA, 25aECM/PLGA and 50aECM/PLGA, respectively. Meanwhile, microspheres with a mass fraction of 25% collagen were prepared, denoted as 25Col/PLGA. The feed ratio is shown in Table 1. Then the viscosity of each suspension was tested by viscometer. Afterward, the resulting aECM/PLGA suspension was drawn into a standard plastic syringe (2 ml). The extrusion speed of the extruder and electric voltage for fabricating the microspheres were maintained at 0.05 cm min^−1^ and 7.0 kV, respectively. The collected aECM/PLGA microspheres were washed by excess distilled water three times and immersed in distilled water 8 h to remove NMP completely, and then lyophilized for 12 h for further investigation.

**Table 1.** Fabrication of aECM/PLGA microspheres with different feed ratios

	1aECM/PLGA	5aECM/PLGA	10aECM/PLGA	25aECM/PLGA	50aECM/PLGA	25Col/PLGA
NMP/mL	10	10	10	10	10	10
PLGA/g	0.7	0.7	0.7	0.7	0.7	0.7
aECM/g	0.007	0.037	0.077	0.23	0.7	0.23

### Morphology characterization

The diameter of the microspheres was measured by grain size shape instrument R-2000 (M.I.P Technology (Changzhou) Co., Ltd). The resultant microspheres were put into the liquid nitrogen to quick-freeze, and cut the microspheres by sharp blade to display the cross-section of microspheres. Then put the frozen microspheres in the freezing dryer (FD-1D-50, Beijing BYK Co., Ltd) for 3 h. Using field emission scanning electron microscopy (SEM, Hitachi S3000 N) to observe the surface and inner structure of the microspheres with platinum spraying on the microspheres.

### Sirius red staining of microspheres

Collagen is part of the ECM proteins that make up the connective tissue matrix. Because collagen is alkaline, it can bind to acidic dyes such as Sirius red, which can be clearly observed under a light microscope [51]. In order to visually observe the distribution of aECM on the surface of the microspheres, the microspheres were stained with Sirius red. The microspheres were rinsed with PBS for three times. Then add the Sirius red dye solution to the microspheres until submersion all the sample and keep 2–3 min, remove the dye and wash three times by PBS. Lastly, the microspheres were observed by light microscope.

### Cell adhesion and proliferation on microspheres

Mouse MC3T3-E1 cells were cultured with DMEM supplemented with 10% FBS, 63 mg/l penicillin and 100 mg/l streptomycin under a humidified atmosphere of 95% air and 5% CO_2_ at 37°C. The culture medium was refreshed every 2 days. Then the microspheres were put into 48-well plates and then immersed in 75% alcohol and exposed to a UV lamp for 2 h, then the alcohol was removed in a sterile environment and washed three times with PBS to completely remove the alcohol. MC3T3-E1 cells were seeded on microspheres at a density of 1 × 10^4^ cells per 500 μl medium per well. After 1, 3 and 7 days of culture, CCK-8 method was utilized to detect the performance of cells on different microspheres. Calcein-AM/PI staining was used to evaluate the cell viability. The cell–microsphere samples were washed with sterile PBS for three times. Then mixing calcein-AM and PI in sterile PBS which the ratio is 2.5 ml PBS, 5 μl calcein-AM and 8 μl PI, and then incubate in the dyeing solution for 15 min at 37°C. The staining results of the samples were observed by inverted fluorescence microscope (Nikon, TE-2000U, Japan).

### Osteogenic differentiation of MC3T3-E1 cells on microspheres

ALP activity was investigated by ALP staining and quantitative kit on Days 7 and 14. The procedure of ALP staining experiment is as follows. Four percent PFA was utilized to fix the sample at room temperature for 20 min, and rinsed with PBS three times. Then the NBT/BCIP ALP staining kit is operated according to the operational protocol. The microspheres and staining solution were incubated at room temperature until color development and observed by light microscopy. ALP quantitative test is performed by p-nitrophenol phosphate assay (pNPP) via ALP assay kit. The cells were rinsed three times with PBS and then lysed with RIPA lysis buffer. The samples obtained after lysed were repeatedly frozen at −80°C and thawed three times at 20°C. After centrifugation of the cell lysates at 1000 g for 10 min, the supernatant was added to the pNPP substrate solution (for ALP assay) and the diquinolinic acid solution (BCA for total protein assay), respectively, and then incubated at 37°C for 30 min. The absorbance of pNPP and BCA solutions at 405 and 562 nm was determined by multifunctional enzyme labeler. The relative change of ALP activity was calculated based on OD405/OD562 ratio.

Calcium deposition of MC3T3-E1 cells on biomimetic bioactive microspheres was assessed by using AR staining (ARS) at 14 and 21 days. First, the sample was fixed with 4% PFA for 20 min, and then washed three times with PBS. Then, 1%AR solution was added to the sample, incubated at 37°C for 1–2 min, taken out and observed the dyeing effect. When the dark orange-red stain was presented, the dye solution could be absorbed and washed with PBS, and then observed and photographed under a type microscope. After observation, 10% CPC solution was added and treated at 37°C for 1 h. Calcium was then quantified via measuring absorbance at 540 nm using an enzyme-labeled instrument.

### Quantitative real-time PCR

MC3T3-E1 cells were seeded at 6-well plate and the density is 2 × 10^5^ cells per 2 ml medium per well. The expression of four osteogenic genes, including runt-related transcription factor 2 (Runx2), osteopontin (OPN) and osteocalcin (OCN), as well as collagen-I (Col-I), were analyzed by real-time PCR to investigate the osteogenic effect of bionic microspheres at 14 and 21 days. The cells were digested from the material using trypsin and the cells were cleaved using the TRIzol reagent and the RNA was extracted according to the reagent protocol. Then the purity and concentration of RNA were detected by Nanodrop Plates Reader (Infinite M200, Tecan, Switzerland). The resulting RNA was reversed transcribed using the PrimeScript RT Reagent Kit with the gDNA Eraser on a PCR instrument. The cDNA obtained by reverse transcription was analyzed by real-time PCR using the Mx3005P real-time quantitative PCR System (Agilent Technologies Inc., USA), as follows: initial heating at 95°C for 30 s, followed by 40 cycles at 95°C for 5 s and 56°C for 34 s, ended by 95°C for 15 s, 56°C for 60 s and 95°C for 15 s. Gene expression was quantized by SYBR Premix Ex Taq RR420A. Gene-specific primers of glyceraldehyde-3-phosphate dehydrogenase (GAPDH), Runx2, OPN, OCN and Col-I were designed by Comate Bioscience Co., Ltd (China) and shown in Table 2.

**Table 2.** Sequences of primers for the qRT-PCR

Gene	Forward primer sequence	Reverse primer sequence
RUNX2	5-GCCCTCATCCTTCACTCCAAG-3′t	5-GGTCAGTCAGTGCCTTTCCTC-3′t
OPN	5-TCAGGACAACAACGGAAAGGG-3′t	5-GGAACTTGCTTGACTATCGATCAC-3′
OCN	5-AAGCAGGAGGGCAATAAGGT-3′t	5-TTTGTAGGCGGTCTTCAAGC-3′t
Col-1	5-CGCTGGCAAGAATGGCGATC-3′t	5-ATGCCTCTGTCACCTTGTTCG-3′t
GAPDH	5-CAACCTGGTCCTCAGTGTAGC-3′C	5-CGTGCCGCCTGGAGAAACCTGCC-3′3

All data were normalized to GAPDH expression. The gene expression levels were obtained using the threshold cycles (Ct). Relative transcript quantities were calculated by the ΔΔCt method according to our previous work.

### *In vivo* evaluations based on rat calvarial defect model

Animal experiments have been approved by the ethical committee of Changchun Institute of Applied Chemistry Chinese Academy of Sciences and confirm compliance with all relevant ethical regulations (20220089). Wistar rats (female, 12 weeks, body weight: 250–300 g) were supplied by Liaoning Changsheng Biotechnology Co., Ltd (Liaoning, China). Rats were anesthetized with a subcutaneous injection of pentobarbital. During surgery, the hair on the dorsum of the skull was shaved and treated aseptically. An approximately 20 mm sagittal incision was made on the scalp of the rat to expose the periosteum of the skull. Two full-thickness skull defects, 5 mm in diameter, were slowly drilled with a dental drill under continuous irrigation with 0.9% saline to prevent local overheating, as marked by the hermetically and sagittal sutures. As shown in Figure 8, the microspheres were then implanted into the defect region. The incision was sutured with 4.0 absorbable silk suture after operation. Antibiotic therapy (penicillin 80 000 IU/day) was given for 5 days after operation. All rats were housed at room temperature and monitored daily for potential complications or abnormal behavior. All rats were randomly divided into five groups, including blank control, PLGA microsphere, 25aECM/PLGA microsphere, 50aECM/PLGA microsphere and 25Col/PLGA microsphere, with four parallel samples in each group.

Micro-CT (SkyScan 1172, Bruker) was used at week 0, week 4 and week 8 to evaluate the repair effect of the skull defect. The raw images obtained by scanning were transcoded using NRecon software for subsequent 3D reconstruction, and Ctvox software was used for 3D reconstruction. Bone volume relative to tissue volume (BV/TV), trabecular number (Tb.N), trabecular thickness (Tb.Th) and trabecular distance (Tb.Sp) were quantified by CTAn software. Then the surgical site of the skull was taken for histological observation. Samples were decalcified with 10% EDTA solution (pH 7.0) for 28 days, dehydrated in ethanol solution with gradient concentration and embedded paraffin sections. The prepared sample was sliced with a microtome (Leica EG 1160, Germany) in a direction perpendicular to the coronal plane. Hematoxylin–eosin (H&E) and Masson trichromatic staining were used to observe the cell and tissue growth in the repaired area.

### Statistical analysis

The data were analyzed using Origin 2019 and are presented as the mean ± standard deviation. The statistic difference was evaluated by variance analysis (ANOVA one-way, Origin 2019). A value of *P *<* *0.05 was regarded as statistically significant.

## Results and discussion

### Effects of solution concentration

**Figure 1.**
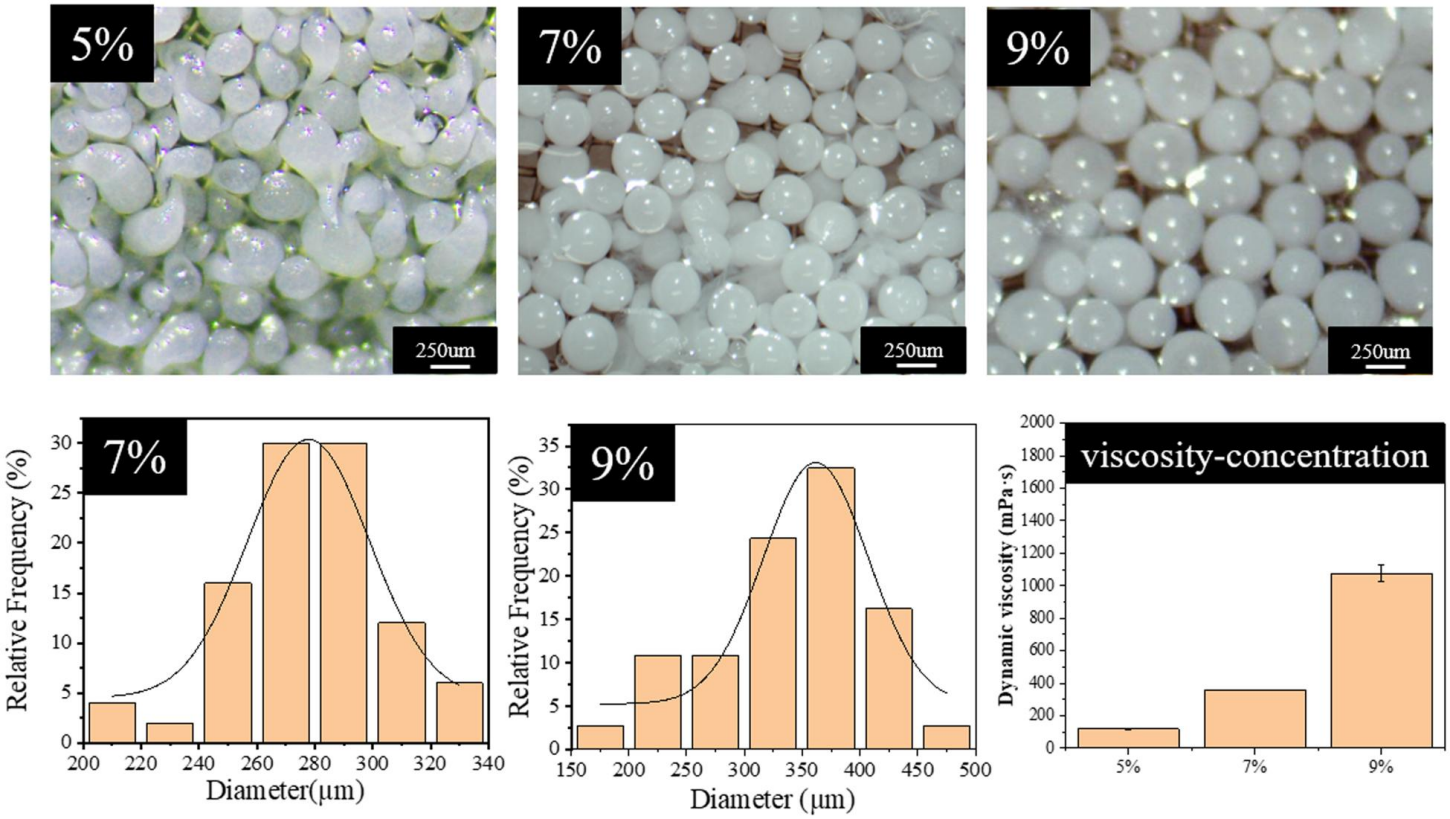
The morphology of microspheres fabricated at the concentration of 5%, 7%, 9% of PLGA and all the length of the scale of 250 μm. The statistical size distribution of microspheres 7% group and 9% group. *N* = 35. The dynamic viscosity of PLGA/NMP solutions at different concentrations.

As Figure 1 shows, when the concentration was 5% of PLGA, the microspheres were irregularly spherical and comet shaped with small tails. The experimental results were in agreement with the reported literature which means the polymer droplet with the too-low viscosity produces a tailing when solidified into a pellet in a reversed-phase solvent [52]. As the concentration increased, the microspheres became regular sphere. Besides, the diameter increased with the concentration increase. The average diameters of 7% group and 9% group are 271.04 ± 34.41 and 341.24 ± 63.82 μm, respectively. As the concentration increases, the range of size distribution becomes wider. The microspheres existed small tails at the concentration of 5%. The reason may the viscosity is too low and the droplets were out of shape. Li *et al.* [30] observed this phenomenon while preparing polycarbonate microspheres. And Lin *et al.* [52] reported this phenomenon while preparing alginate microspheres those results show in the process of polymer solution using antisolvent replacement, too low concentration will lead to irregular sphericity and produce tailing phenomenon. As the result of the dynamic viscosity of PLGA/NMP solutions at different concentrations showed, the dynamic viscosity varied from concentration sharply. The dynamic viscosity of 5% and 7% PLGA/NMP solution were 117 and 355 mPa⋅s, respectively. The dynamic viscosity of 7% PLGA/NMP solution increased to 1073.6 mPa⋅s. As the increase of the concentration, the viscosity of the solution increases, the strength of the droplets increases, the spherical shape of drop is more stable, and it is easier to form droplets with good sphericity which is also consistent with those reported in the literature [30, 52]. As the concentration of 7% of PLGA can fabricate a more spherical and uniform size microspheres. Herein, the next fabrication process would use the concentration at 7% of PLGA.

### Effects of voltage

**Figure 2.**
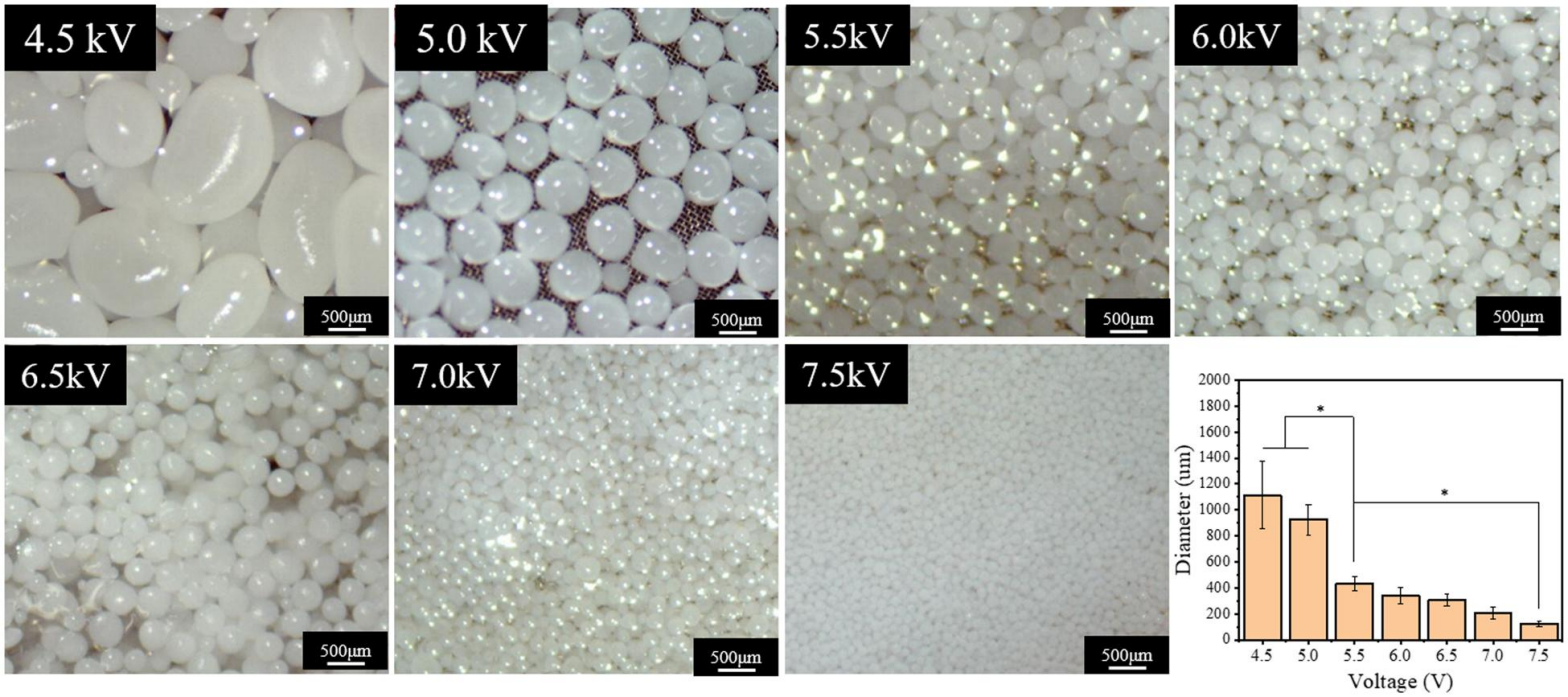
Appearance of PLGA microspheres prepared at different voltages (5.5, 6.0, 6.5, 7.0, 6.5 kV), and the average diameters of different microspheres. All the scale bars are 500 μm. *N* = 25 *P < 0.05.

The effect of voltage from 4.5 to 7.5 kV on the diameter of PLGA microspheres was investigated. Figure 2 showed the photos and variation in microspheres size for voltage from 4.5 to 7.5 kV. As the voltage of electrostatic field increased, the size of microspheres decreased remarkably. For the voltages at 4.5 and 5.5 kV, it was found that the diameters of microspheres were 1112.30 ± 259.955 and 924.18 ± 115.4 μm, respectively. Because the electrostatic force applied on the drop was too low, the drop could not spray but only fall down until the gravity was greater than the surface extension. As reported by Andreu *et al.* [53], when the voltage increase, the electric charge accumulate and the solution breaks up and splits into small drops because of the effect of electric field force. The diameter of the drop hence becomes smaller. The diameter of microspheres was distributed between 433.23 ± 55.92 and 123.53 ± 19.87 μm with voltages between 5.5 and 7.5 kV. As the literature reported that under the optimal curvature of the bone repair microspheres, F-actin cytoskeleton formation, nuclear deformation and Lamin A expression were significantly enhanced [25]. Therefore, the microspheres fabricated at the voltage of 7.0 kV meet the requirements best and the corresponding size is 205.05 ± 47.30 μm.

### Fabrication and morphology of microspheres

**Figure 3.**
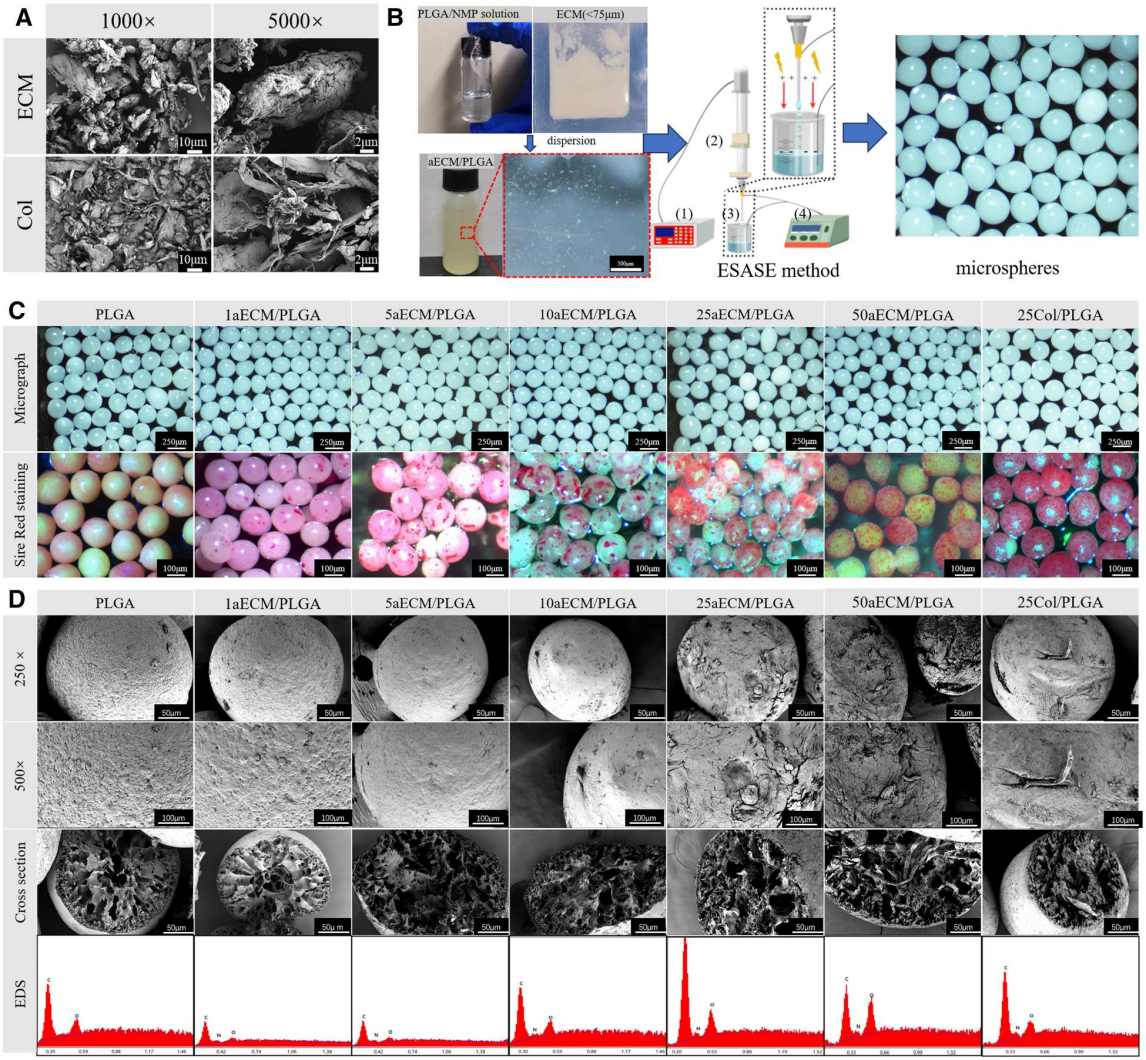
(**A**) SEM of aECM and collagen. (**B**) Rapid fabrication of microspheres, (1) stepper motor controller, (2) extruder, (3) collecting device, (4) high voltage electrostatic generator. (**C**) Micrographs and the staining of sire red of different microspheres containing different amounts of aECM. The bars of the micrographs are 250 μm and the bars of sire red staining are 100 μm. (**D**) Morphology and cross-section of microspheres and EDS of microspheres. The bars of morphology of 250× and cross-section are 50 μm. The bars of morphology of 500× are 100 μm.

Using the aforementioned parameters of optimal PLGA concentration of 7% and optimal voltage of 7 kV, different microspheres containing different amounts of aECM were fabricated by ESASE. The microstructure of aECM and collagen are shown in Figure 3A, the aECM is granular form and the collagen is mainly fibrous form. Figure 3B shows the fabrication of microspheres, the aECM powder was uniformly dispersed in PLGA/NMP solution and then fabricated into microspheres by ESASE method. It only takes about 30 min for 1 g aECM/PLGA microspheres to be solidified from aECM powder to microspheres. The generation of derived aECM on the surface of microspheres by decellularization technology generally requires the cells to be cultured on the surface of the material for 10–14 days in advance, which is very time-consuming [54–56]. Moreover, the decellularization process is complicated and cumbersome [57]. The spray drying method also takes at least 1.5 h and the solvent evaporation method takes 5 h [58]. In contrast, ESASE is a rapid method for the preparation of aECM/PLGA microspheres. From Figure 3C, as the adding of the aECM, the microspheres could still keep the spherical shape. And the size can be controlled uniformly. The size of different aECM/PLGA was shown in Supplementary Figure S1. The diameter of microspheres was positively related to the dynamic viscosity. The minimum diameter among groups was PLGA microspheres and the maximum diameter was 50 aECM/PLGA microspheres. The values of minimum and maximum diameter were 202.21 ± 15.08 and 212.72 ± 16.72 μm, respectively. All the groups meet the requirement of optimal diameter. As Supplementary Figure S2 shows, the dynamic viscosity of aECM/PLGA/NMP suspension varied from 355 to 634 mPa⋅s. The Sirius red staining has specific reaction of collage of the aECM and can dye the aECM red. As Figure 3C shows, the aECM was exposed on the surface of the microspheres. As the increased of the content of the aECM, the more aECM was exposed. As Figure 3D shows, the PLGA, 1aECM/PLGA and 5aECM/PLGA microspheres had smooth surface, and there were great number of pores at the inner of the microsphere. The size of the pore decreased from the center to the surface. This phenomenon was consistent with that reported in the literature by Yan *et al.* [1]. The main reason is that the PLGA/NMP drop went into the ethanol solution and then the NMP was absorbed by the ethanol solution while the PLGA precipitated and left a lot of channels for NMP to dissolve into the solution and then the channels become pores when the microsphere lyophilized. On the other hand, with the increase of aECM content, the surface of the microspheres became rough (25–50 aECM/PLGA group and 25Col/PLGA group). And 1–10 aECM/PLGA group kept smooth. The EDS spectrum of the microspheres section indicates the existence of the aECM, as the signal of N was found in the spectrum. From the Sirus red staining of microspheres and the EDS of cross-section of microspheres, the aECM could be observed both at the surface and inner of the microspheres. The rough surfaces exposed by aECM indicate that the biomimetic microspheres were successfully fabricated.

### Cell proliferation and fluorescence cell-staining results

**Figure 4.**
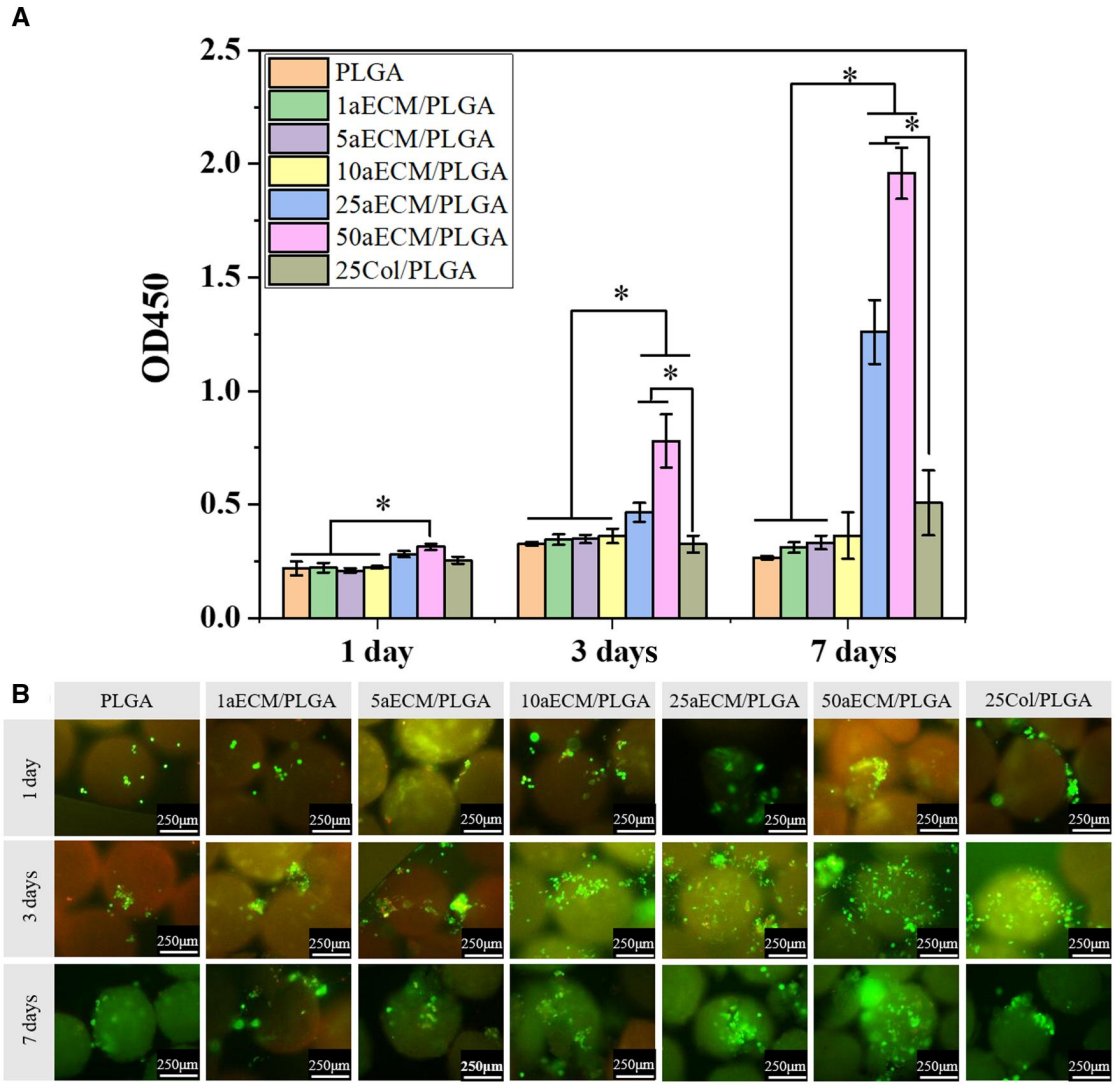
(**A**) CCK-8 test of the proliferation of MC3T3-E1 on the PLGA and aECM/PLGA microspheres for 1, 3 and 7 days; *n* = 3. *P < 0.05. (**B**) Micrographs of the adhesion and growth of MC3T3-E1 on the PLGA and aECM/PLGA microspheres at 1, 3 and 7 days stained with calcein-AM/PI. All scale bars are 250 μm.

In order to observe the cell proliferation, using CCK-8 to evaluate the cell condition. The calcein-AM/PI staining was carried out to evaluate cell viability and the result was shown in Figure 4. From Figure 4A, no difference was observed in terms of cell adhesion for all the tested conditions at Day 1. However, at Days 3 and 7, the 25–50 aECM/PLGA showed a significant increase of MC3T3-E1 cells, as compared to PLGA and 1–10 aECM/PLGA groups. These results were confirmed by calcein/PI depicted in Figure 4B showing a higher pattern of cells adhered at the surface of the microspheres on these groups at Days 3 and 7. At Day 7, the increase of cell proliferation was highly pronounced, especially on the 50ECM/PLGA group, which means the ECM and induced roughness increase the cell adhesion and proliferation. The amount of aECM has significant effect of the cell adhesion. Pathak *et al.* [47] reported that the collagen can also help the cell adhesion because the collagen is a typical cell adhesion a large number of arginine-gly-aspartic acid (RGD) sequences distributed on its molecular chain provide an active site for the adhesion of cells. Zhou *et al.* [15] proved that aECM can help the adhesion and proliferation of cell as the aECM contains adhesion proteins and offers microenvironment of cells. Diener *et al.* [59] verified that rough surface was more conducive to cell adhesion the rough surface is conducive to local adhesion formation, integrin aggregation and local adhesion mobility. In this study, when the aECM content lower than 5%, the microsphere is smooth which results the poor cell adhesion. As the content of aECM increases, with the rough surface of biomimetic microsphere and the more aECM exposed on the surface of microspheres, the ability of cell adhesion and proliferation was enhanced. Besides, the results of 25Col/PLGA showed lower values of cell proliferation compared to 25aECM/PLGA. This result may be caused by the effect of the remaining components of the ECM apart from collagen.

### The alkaline phosphatase activity evaluation

**Figure 5.**
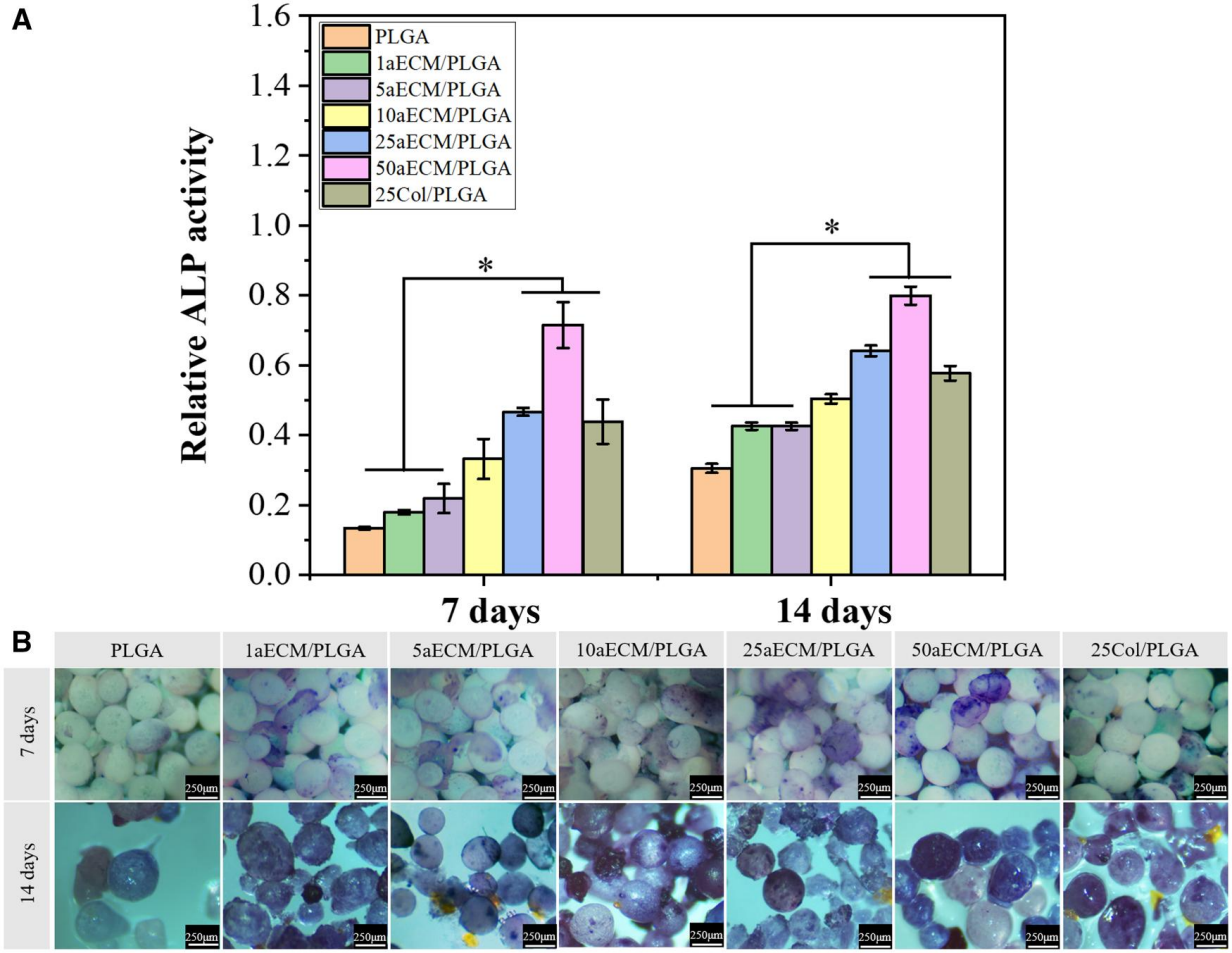
(**A**) The activity of corresponding ALP quantitative evaluation analysis. The scale bars are 250 μm. *P <* *0.05. (**B**) The ALP staining of MC3T3-E1 cells cultured on different microspheres for 7 and 14 days.

ALP activity increased significantly before osteoblast differentiation and is an important indicator of osteogenic differentiation [41]. The aECM has the effect of providing ALP activity of cells and inducing differentiation into osteoblasts [15, 60]. Figure 5A showed the value of ALP activity of aECM/PLGA microspheres was higher than that on PLGA microspheres after 7 and 14 days of culture which was confirmed by the staining results. It indicated that the aECM also increased the activity of MC3T3-E1 and showed the potential to differentiate into osteoblasts. Besides, the difference between PLGA and 50aECM/PLGA microspheres was statistically significant. At 7 and 14 days of culture, the ALP activity of 50aECM/PLGA microspheres was 5.3 and 2.6 times higher than that of PLGA microspheres, respectively. Figure 5B showed the ALP staining of MC3T3-E1s on the surfaces of the biomimetic microspheres. The staining of 50aECM/PLGA microspheres appeared darker than the microspheres which the content of aECM was lower than 5% at 7 days. After 14 days of culture, the staining of each group deepened, especially 50aECM/PLGA group which was in accordance with the quantitative result. The results indicated that the composition of aECM and PLGA can effectively increase the ALP activity of MC3T3-E1 and may promote the enhancement of bone formation ability *in vitro*.

### Alizarin red staining and calcium deposits

**Figure 6.**
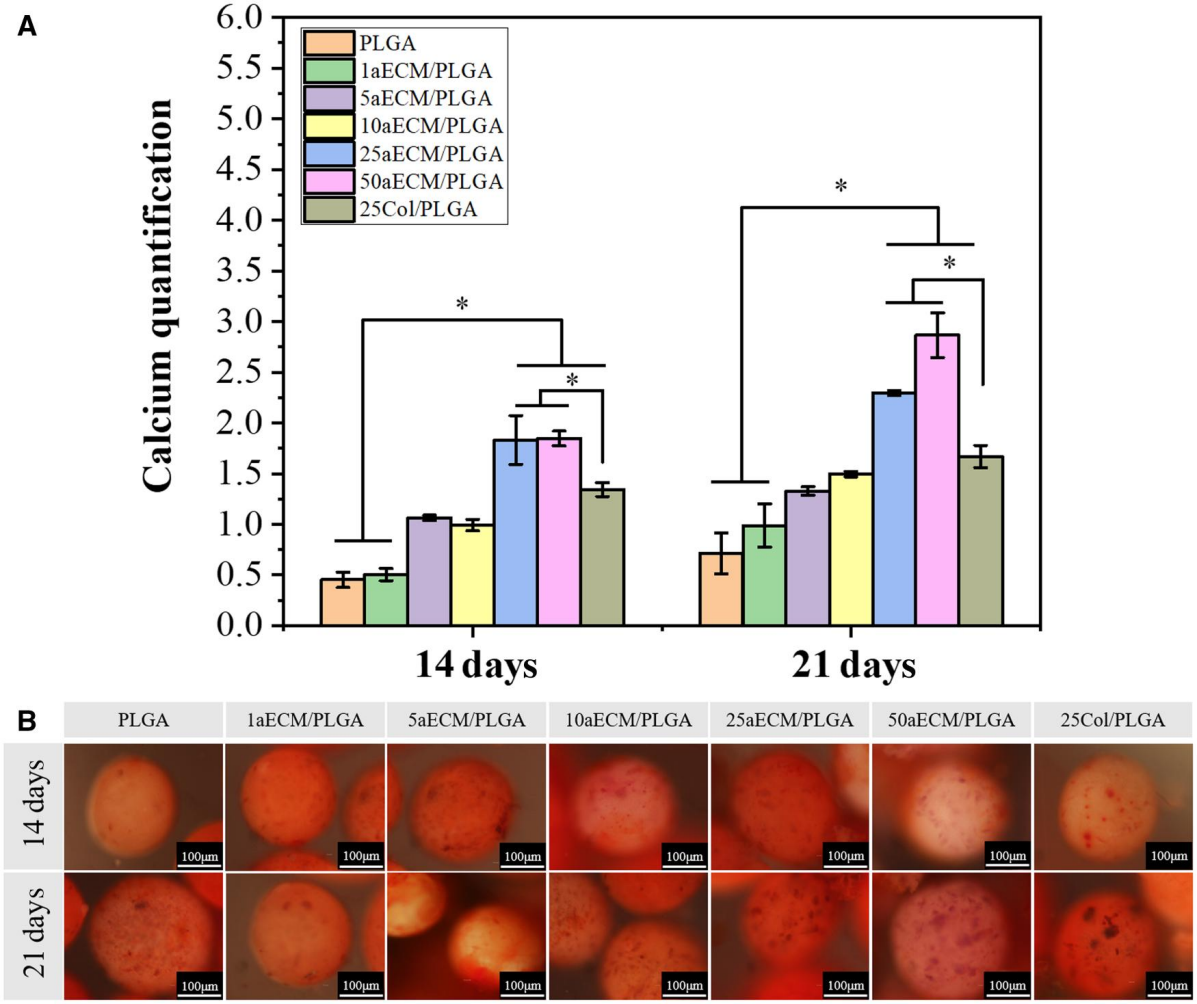
(**A**) Corresponding quantitative evaluation of calcium content mineral deposition of MC3T3-E1 cells cultured on different microspheres after 14 and 21 days. *P < 0.05 (**B**) ARS of MC3T3-E1 cells cultured on different microspheres after differentiation for 14 and 21 days. All the bars are 100 μm.

Calcium deposition is an important indicator of osteogenic differentiation. ARS and calcium quantification are effective methods to characterize the osteogenic differentiation of preosteoblasts on different microspheres [1]. The 10% CPC was used to extract ARS nodules, and calcium deposition was measured so as the bone mineralization ability of different microspheres was quantitatively evaluated. As shown in Figure 6A, the total calcium content of the 50aECM/PLGA group was the highest at 14 and 21 days. At 14 days, the 25aECM/PLGA and 50aECM/PLGA groups were significantly higher than that of PLGA and 1aECM/PLGA groups. There was slightly difference between 25aECM/PLGA and 50aECM/PLGA at 14 days, while the calcium content of 50aECM/PLGA was significantly higher than 25aECM/PLGA groups at 21 days. The calcium content in each group increases with the increase of the content of ECM at 21 days which means the content of aECM affects the calcium deposition. The calcium content of 25aECM/PLGA and 50aECM/PLGA groups was significantly higher than that of other groups at 21 days. Different ECM species have different influence on osteogenic differentiation [60]. In this study, it was also observed that the calcium content of 25aECM/PLGA group was higher than that of 25Col/PLGA group. Figure 6B showed that PLGA, 1aECM/PLGA, 5aECM/PLGA and 10aECM/PLGA microspheres had mild staining. However, MC3T3-E1 cells with 25aECM/PLGA, 50aECM/PLGA and 25Col/PLGA microspheres all showed strong red nodule staining after 14 and 21 days, indicated that calcium deposition induced by high aECM/PLGA microspheres was better than that induced by low aECM/PLGA microspheres. A large number of AR nodules were distributed on aECM/PLGA microspheres, confirming that microspheres containing aECM components significantly promoted calcium deposition after 21 days, the difference between groups was similar to that after 14 days. The staining intensity of calcium nodules in the 50aECM/PLGA group was more significant than that in the other groups, and the surface of the microspheres was covered with a large number of calcium staining nodules. Besides, 25aECM/PLGA group showed more calcium staining nodules than 25Col/PLGA group. The above results indicated that 25aECM/PLGA and 50aECM/PLGA microspheres have stronger osteogenic differentiation and calcium deposition ability, and the promotion effect of aECM is better than that of collagen alone.

### Expressions of osteogenesis-related genes

**Figure 7.**
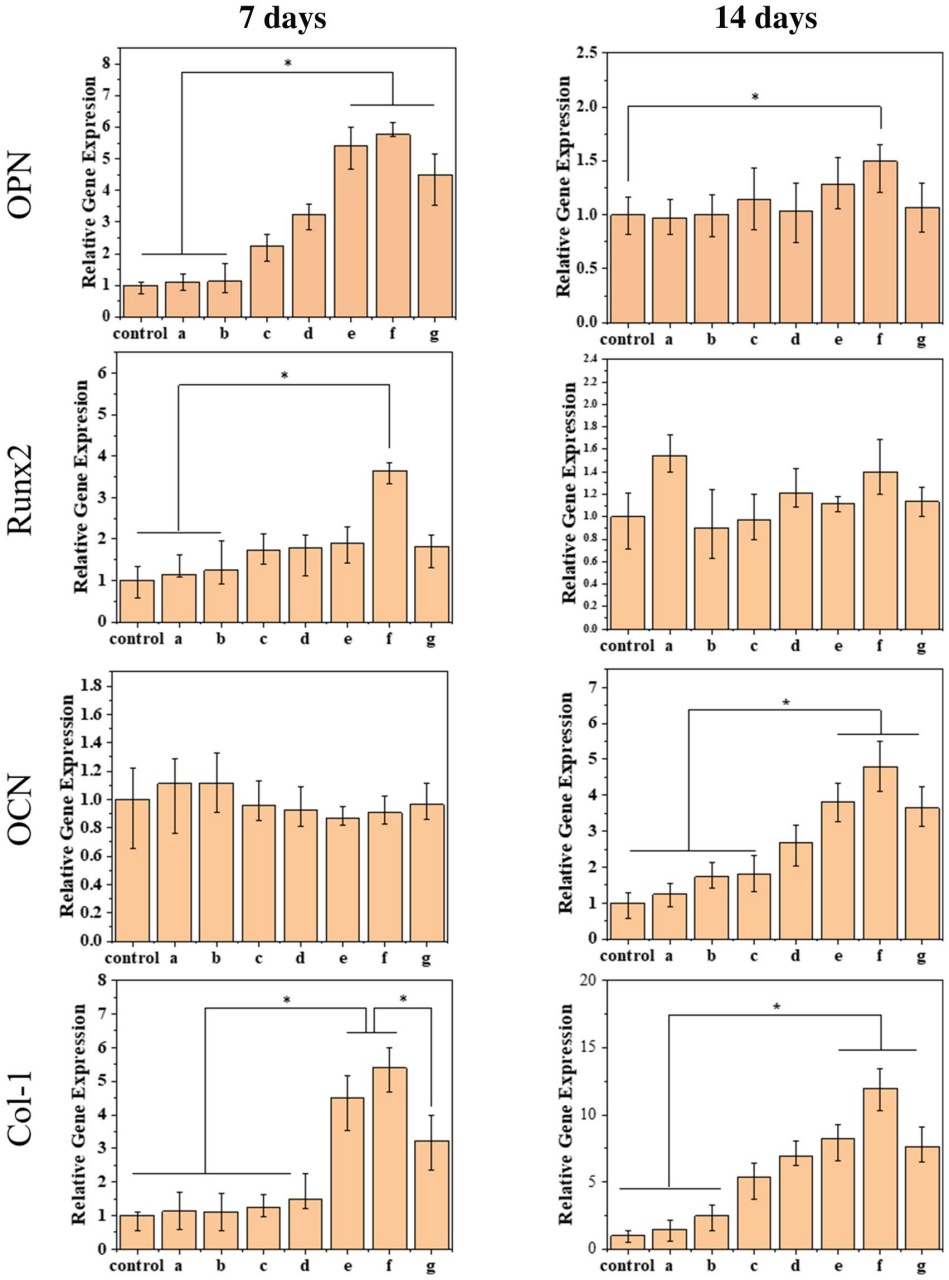
MC3T3-E1 cells were cultured on the different microspheres for 7 and 14 days (control: plate culture, a: PLGA, b: 1aECM/PLGA, c: 5aECM/PLGA, d: 10aECM/PLGA, e: 25aECM/PLGA, f: 50aECM/PLGA, g: 25Col/PLGA). The expression of four osteogenic genes (Runx2, OCN, OPN and col-1) was analyzed by qRT-PCR. The intensity of each gene was normalized to the value of GADPH. *P < 0.05.

The expression levels of four osteogenic-related genes (OPN, Runx2, OCN and Col-I) were analyzed by real-time quantitative PCR in MC3T3-E1s cultured with aECM/PLGA microspheres and the corresponding control PLGA for 7 and 14 days, and the results are summarized in Figure 7. Cell adhesion, proliferation and differentiation are regulated by different genes [61]. *In vitro* experiments showed that aECM/PLGA microspheres could obtain a sufficient number of preosteoblasts and exhibited strong ALP activity and calcium deposition ability. However, the effects on cell differentiation and proliferation after inoculation are unknown. Runx2 was expressed at the early differentiation stage. OPN expression could be observed after the middle stage and OCN express at the late differentiation stage. The Col-I gene is first expressed at the initial stage of ECM production [2, 61]. As shown in Figure 7, after 7 days of culture, the expression levels of Runx2 and OPN in 50aECM/PLGA microspheres group were higher than those in PLGA microspheres group, and the expression of OPN in 25aECM/PLGA, 50aECM/PLGA and 25Col/PLGA microspheres groups were significantly different from those the aECM content was lower than that of 10aECM/PLGA microspheres group. Besides, expression levels of OPN of 25aECM/PLGA microsphere group was higher than the 25Col/PLGA group, indicated that the aECM may have the better regulation than the collagen. The expression levels of OCN on the microspheres which contained a small number of aECM were similar. For Col-I, the 25aECM/PLGA, 50aECM/PLGA and 25Col/PLGA were significantly higher than other groups after 7 days. After 14 days, the difference of Runx2 and OPN between 50aECM/PLGA microspheres group and other groups decreased, and the difference among groups was not statistically significant. However, the OPN expression level in 50aECM/PLGA group was still significantly higher significantly higher than the control with PLGA alone. After 14 days, the expression levels of OCN and Col 1 in each material group were significantly increased. Natural bone matrix is rich in collagen (mainly type I collagen). OCN is seen as a marker of bone formation because it is a specific protein expressed during the maturation of the osteogenic matrix. The results of real-time PCR showed that the expression levels of bone-specific maturation genes OCN and Col-1 in 25aECM/PLGA and 50aECM/PLGA microspheres groups were significantly higher than those in other groups, indicating that 25aECM/PLGA and 50aECM/PLGA microspheres had the ability to promote osteoblast differentiation and bone formation. As the results showed, the Runx2, OPN and Col-1 expressed at the early stage, MC3T3-E1 began to secrete collagen matrix and the expression of Runx2 was up-regulated and mediated osteogenic differentiation. The cells began to differentiate into osteoblasts, and the gene expression level of osteogenic marker protein OPN increased. After 14 days, the expression of Runx2 was almost not obvious, while the expression of OCN, a marker of late development, was significantly up-regulated, and the expression of Col-1 was also up-regulated compared with PLGA group. The osteopromoting effect of aECM/PLGA microspheres was demonstrated at the gene level, which was consistent with the expression program of marker proteins during osteogenesis. By comparing the expression levels of marker genes between the 50aECM/PLGA microspheres group and the 10aECM/PLGA, 5aECM/PLGA, 1aECM/PLGA and PLGA groups, it can be seen that the osteogenesis level of the ECM-rich microspheres was significantly higher than that of the microspheres with low aECM content and without aECM. Thus, the increased gene expression levels on aECM/PLGA microspheres might have resulted from the bioactivities of aECM. As can be seen from the comparison between 25aECM/PLGA microspheres group and 25Col/PLGA microspheres group, it can be seen that the osteogenesis level of the microspheres with multi-component and micro-structure aECM was significantly higher than that of the pure collagen microspheres. This means that aECM can promote osteogenic differentiation of cells more than pure collagen.

### Evaluation of bone regeneration *in vivo*

**Figure 8.**
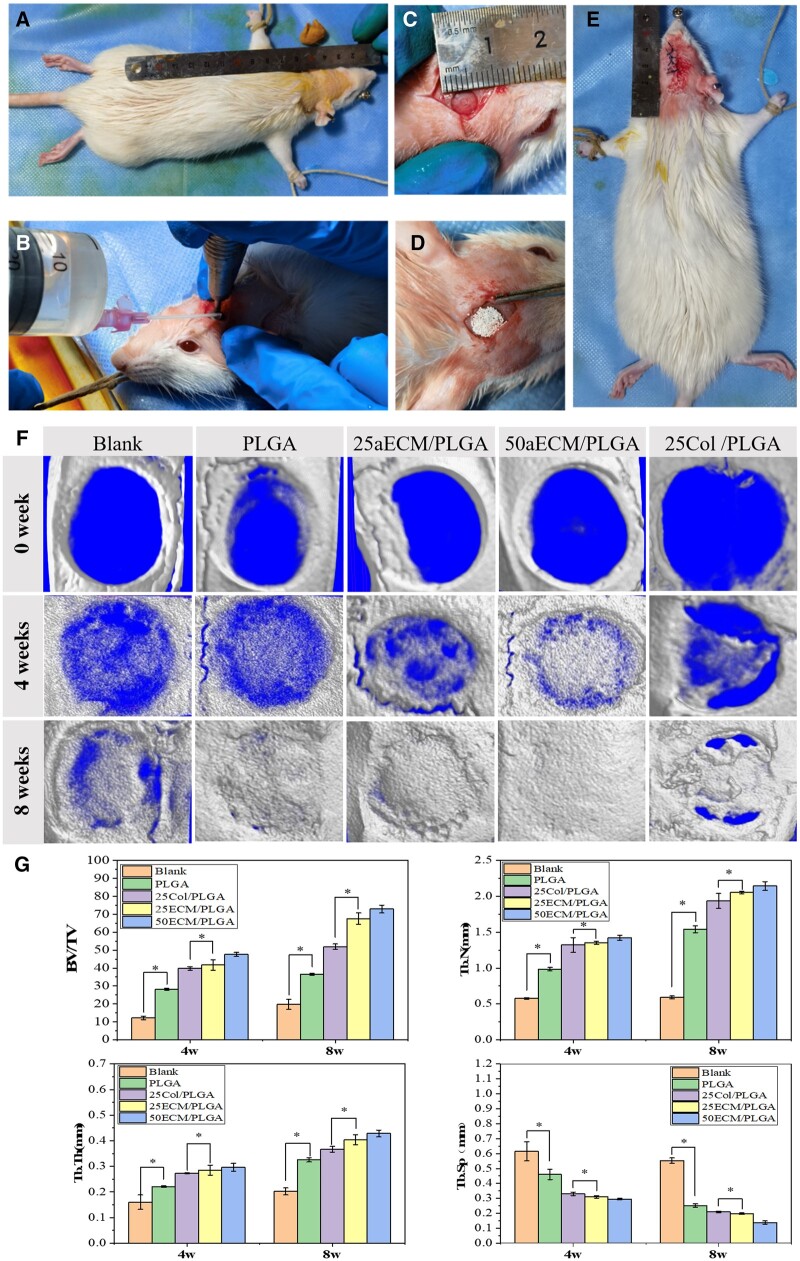
(**A**) Preparation for surgery. (**B**) Perform the surgery. (**C**) The calvarial defects (the defect size is 5 mm). (**D**) The implant of microspheres at the defect. (**E**) Postoperation of rats. (**F**) 3D reconstructed micro-CT images of bone formation at the rat calvarial bone defect areas at 0, 4 and 8 weeks. (**G**) The quantitative micro-CT bone parameters of bone volume/tissue volume (BV/TV) ratio, trabecular number (Tb.N), trabecular thickness (Tb.Th), trabecular separation (Tb.Sp) after 4- and 8-week postimplantation. *P < 0.05.

*In vitro* experiments demonstrated that 50ECM/PLGA microspheres have the best function of promoting cell differentiation into osteoblasts. The effect of microspheres on bone repair was further investigated in rats with calvarial defects. As shown in Figure 8A, the bone defect area could be clearly observed on the reconstructed images. Micro-CT scan of the bone defect was performed immediately after surgery, and the pictures of 0 W showed that the skull of the rat had a circular defect with a diameter of 5 mm. At 4 weeks after surgery, the amount of new bone formation in 50aECM/PLGA group was significantly higher than that in other groups. At 8 weeks after operation, the blank group still had a large unclosed defect and 25Col/PLGA group had less area of unclosed defect. However, the bone defect was almost completely closed by new bone in PLGA, 25aECM/PLGA and 50aECM/PLGA groups. The bone volume/tissue volume (BV/TV) ratio of regenerated bone tissue, trabecular number (Tb.N), trabecular thickness (Tb.Th) and trabecular separation (Tb.Sp) were measured to quantitatively evaluate bone repair efficiency. The results at 4 and 8 weeks after surgery were shown in Figure 8B. As the results showed, the BV/TV of the PLGA group (28.05 ± 0.58%) was 2.33 times higher than that of the blank group (12.17 ± 1.01%) at week 4, and the BV/TV of the PLGA group (36.55 ± 0.61%) was 1.89 times higher than that of the blank group (19.71 ± 2.77%) at week 8. Although PLGA is a bioinert material, it can provide support for osteocyte growth and migration to promote bone repair. The BV/TV of 25aECM/PLGA group (41.67 ± 2.91%) was 1.04 times higher than that of 25Col/PLGA group (39.75 ± 1.02%) at week 4, and the BV/TV of 25aECM/PLGA group (67.55 ± 3.16%) was 1.30 times higher than that of 25Col/PLGA group (51.91 ± 1.54%) at week 8. The addition of bioactive materials greatly promoted the effect of bone repair. At the same time, the promoting effect of aECM was stronger than that of pure collagen. Besides, the value of BV/TV of 50aECM/PLGA was 47.57 ± 1.14%. After 8 weeks, the value of BV/TV of 50aECM/PLGA was 72.92 ± 2.19%. The BV/TV of the 50aECM/PLGA group was significantly higher than that of the other groups. In addition, the values of Tb. Th and Tb.N of the 50aECM/PLGA group were significantly higher than that of the other four groups, and the value of Tb.Sp of 50aECM/PLGA was the lowest among all groups. These results showed that the 50aECM/PLGA group had the best repair effect. Overall, the 3D reconstructed images and quantitative analysis results showed that the microspheres can help the bone repair and the 50aECM/PLGA microspheres had the best bone formation effect. Besides, the repair effect of the microsphere containing aECM was stronger than that of the group containing collagen, which was consistent with the results of *in vitro* cell experiments.

### Histological analysis of repair area

**Figure 9.**
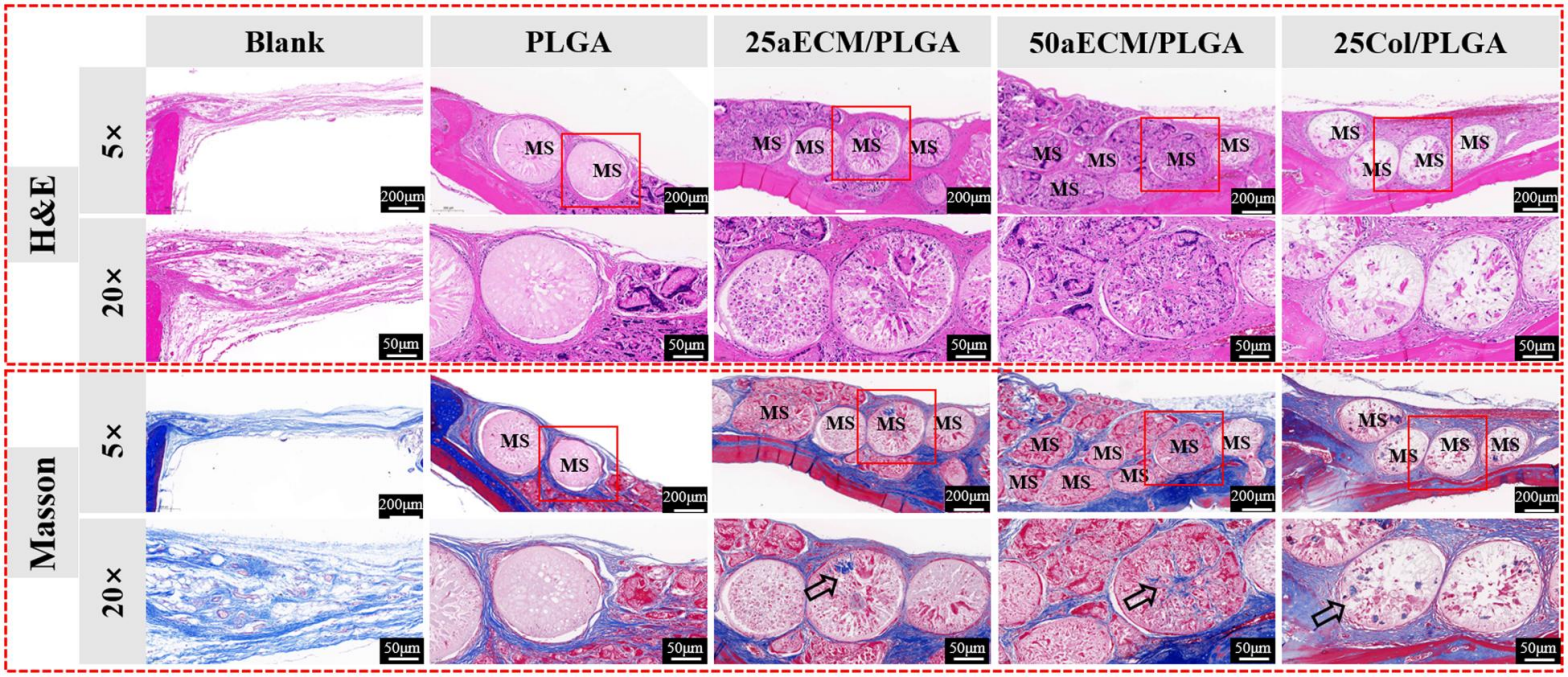
H&E staining and Masson trichrome staining in operative site at 8 weeks. MS, microspheres. The scale bars of 5× and 20× are 200 and 50 μm, respectively.

As Figure 9 shows, after 8 weeks, it can be observed that there were large cracks inside the microspheres, and some microspheres have been damaged, which may be caused by the degradation of microspheres *in vivo*. However, PLGA did not degrade totally, the microspheres still maintained sphericity, and the internal loose and porous structure of the microspheres could be observed clearly. In the picture of H&E staining, the nuclei were stained blue, and cytoplasm and ECM were stained with varying degrees of red. In the blank group, the tissue structure was loose and no typical bone structure appeared. In the PLGA group, there was a certain gap between the tissue and the microspheres, but the aECM/PLGA and Col/PLGA microspheres were closely connected to the surrounding tissue. As an inert material, PLGA has a low affinity with tissues. The addition of aECM and collagen increases the histocompatibility of microspheres. When the microspheres and tissue were observed at a 20-fold field of view, a small number of cells were attached and arranged regularly on the surface of the PLGA microspheres, but no traces of cell and tissue staining were found inside the microspheres. A large number of cells were arranged regularly on the surface of aECM/PLGA microspheres and Col/PLGA microspheres, and cells and tissues were also stained in the interior of the microspheres, and cells infiltrated into the interior of the microspheres, indicating that the cell migration ability in the bioactive microspheres was better. In addition, compared with 25aECM/PLGA group, 50aECM/PLGA group had more internal cells and more stained cytoplasmic and matrix components, indicating that aECM content had an impact on cell migration and growth, and microspheres with higher aECM content had a better effect on cell migration and growth inside the microsphere. It was also observed that compared with 25Col/PLGA group, 25aECM had more cells on the surface and inside, and darker staining color, indicating better cell migration and growth. Masson trichrome staining was used as a specific staining method for collagen fibers to further evaluate the formation and maturation of bone tissue. The collagen tissue is stained blue, and as the collagen tissue matures, the blue color becomes darker. From Figure 9, regular blue collagen bundles were observed between each group of microspheres, and the collagen content in the bone defects filled with 25aECM/PLGA and 50aECM/PLGA microspheres was significantly more than that in the bone defects filled with PLGA microspheres alone, which may be related to the occurrence of osteogenesis. In addition, a large number of mature dense bone tissues were observed in 25aECM/PLGA, 50aECM/PLGA and 25Col/PLGA groups. The 50aECM/PLGA group was more able to attract new tissue to grow between the microspheres, reflecting the possibility of a large amount of new bone formation around the aECM/PLGA microspheres. There was no staining in the microspheres of PLGA group when observed at high magnification. Blue stained tissue which was pointed out by the black arrows was found inside 25aECM/PLGA, 50aECM/PLGA and 25Col/PLGA microspheres, indicating that new collagen was formed inside the microspheres. At the bone defect area, the recruitment and migration of osteoblasts occur first [62–64]. aECM provides a suitable microenvironment for cell growth and adhesion [65]. When osteoblasts were recruited and migrated to the defect area, the aECM exposed on 25aECM/PLGA and 50aECM/PLGA microspheres could effectively adhere to cells and promote cell proliferation. As the aECM/PLGA microspheres can promote calcium deposition and induce cells to differentiate into osteoblasts as shown in Figures 6 and 7. The osteogenesis of the defect was significantly enhanced. Mineralization and collagen deposition during bone repair are the basis of bone repair [66]. The collagen deposition around the 50aECM/PLGA microspheres was evident from H&E staining and Masson staining, indicating better osteogenesis. The repair effect of 25aECM/PLGA microspheres was better than that of 25Col/PLGA microspheres. This may indicate that the aECM used in this study is more effective for osteogenesis than the single collagen component. Taken together with the previous results, the *in vivo* experiments can prove that aECM/PLGA microspheres have a good effect of promoting bone formation, which is a promising material for bone tissue engineering.

**Figure 10.**
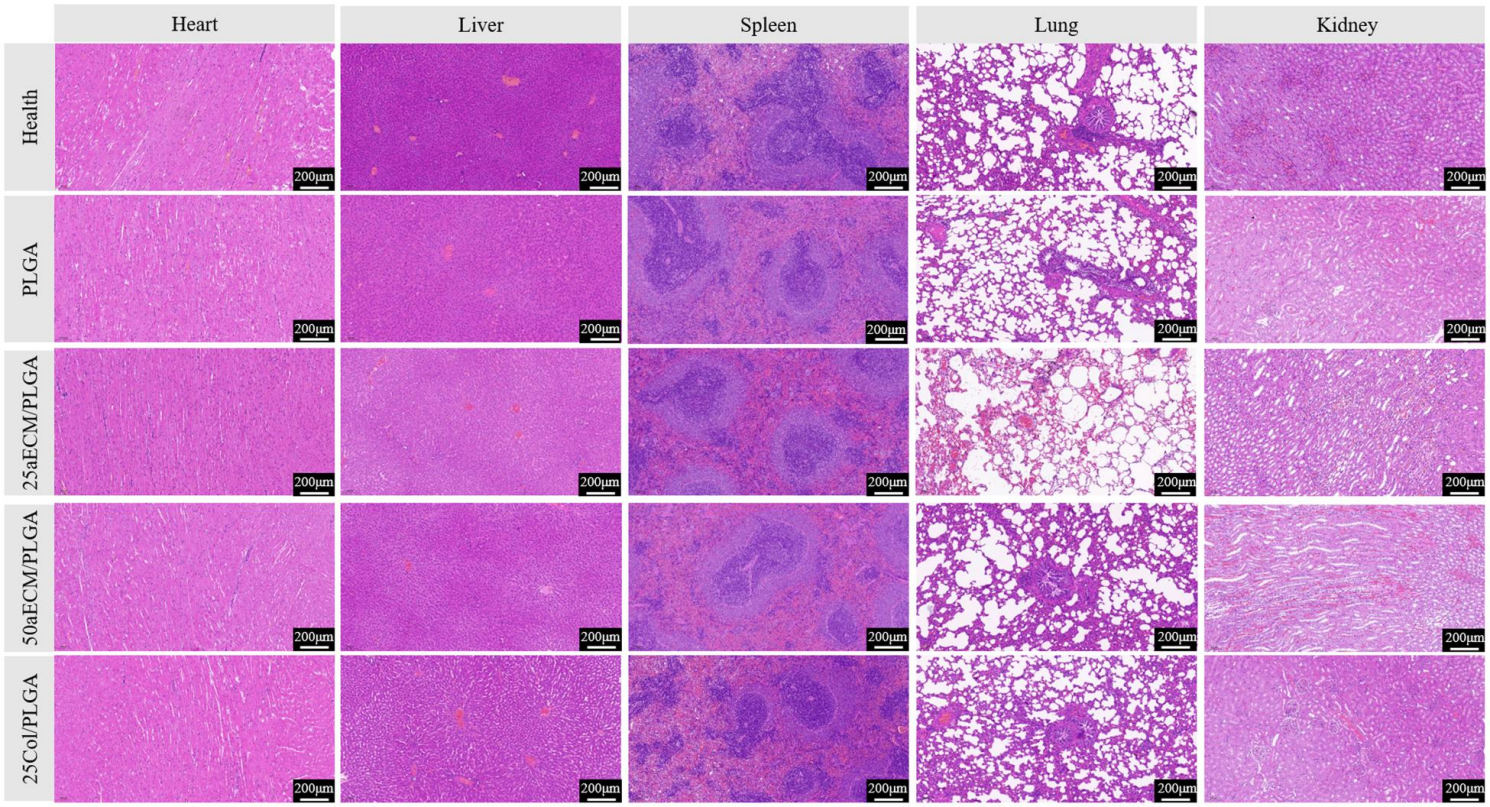
H&E staining of the heart, liver, spleen, lung and kidney. The scale bars are 200 μm.

The biosafety of the microspheres was further evaluated. As Figure 10 shows, the heart tissue structure of all groups shows a typical myocardial cell arrangement, has the obvious fiber structure, closely connected between myocardial cells, form crisscross mesh structure. In addition, cell morphology showed that mouse cardiomyocytes were long strips with branches and processes, and obvious horizontal striation and organ structures such as mitochondria could be seen in the cells. When the liver tissue was examined by section of all the groups, it was observed that the central vein was in the middle of each hepatic lobule, and the hepatocytes were radially arranged around the central vein. The essence of spleen red pulp and white pulp boundary is clear and there is no difference between all groups. As for the analysis of lung, the alveolar septum was rich in capillaries, connective tissue and monocytes. The sections of the kidney tissue of all groups showed that the glomeruli, tubules and other structures were clearly visible without obvious abnormalities or normal morphology of the lesion cells, and there was no obvious irregular shape of the nucleus and numbers of cytoplasm. The tissue structure was intact, and the glomeruli, tubules and other tissues in the sections were structurally intact without breakage or loss. The results showed that none of the microspheres had toxic effects on major organs *in vivo*.

## Conclusions

In the present study, bioactive PLGA microspheres containing natural porcine dermal aECM were rapidly fabricated using ESASE method. The prepared microspheres showed uniform morphology and the exposed aECM creates a bionic surface, which can supply microenvironment for cell adhesion and cell growth. Besides, the *in vitro* experiment showed ALP activity of aECM/PLGA microspheres was enhanced and the ALP activity of 50aECM/PLGA microspheres was 2.6 times than that of PLGA microspheres. And 50aECM/PLGA microspheres have stronger calcium deposition ability than PLGA microspheres which showed that the aECM/PLGA has the function of osteogenic induction differentiation. The results of micro-CT and histology analysis in animal test showed that aECM/PLGA microspheres exhibited better rat skull healing effect and lower immunogenicity at 4 and 8 weeks postoperatively and the value of BV/TV of 50aECM/PLGA were 47.57 ± 1.14% and 72.92 ± 2.19%, respectively. From the above results, it is concluded that the biodegradable PLGA microspheres containing aECM are a kind of promising novel materials for bone tissue engineering.

## Supplementary Material

rbae099_Supplementary_Data
