# Supplementary material for: Rapid fabrication of biomimetic PLGA microsphere incorporated with natural porcine dermal aECM for bone regeneration
Source: Regen Biomater. 2024 Aug 26;11:rbae099. doi: 10.1093/rb/rbae099 (PMC11512121; doi:10.1093/rb/rbae099)
Supplement: rbae099_Supplementary_Data [file rbae099_supplementary_data.zip › supporting material.docx]

##### Rapid Fabrication of Biomimetic PLGA microsphere incorporated with natural porcine dermal ECM for bone regeneration

Xiaosong Zhouab, Min Guoa, Zongliang Wanga*, Yu Wanga*, Peibiao Zhangab*.

a Key Laboratory of Polymer Ecomaterials, Changchun Institute of Applied Chemistry, Chinese Academy of Sciences, 5625 Renmin Street, Changchun 130022, P. R. China.

b School of Applied Chemistry and Engineering, University of Science and Technology of China, 96 Jinzhai Road, Hefei, Anhui 230026, P. R. China.

* Corresponding author: Peibiao Zhang, Yu Wang, Zongliang Wang

E-mail address: zhangpb@ciac.ac.cn (P. Zhang), wydna@ciac.ac.cn (Y. Wang), wangzl@ciac.ac.cn (Z.Wang)

Tel.: +86-431-85262058;

Fax: +86-431-85262058.

**Figure S1.** The average diameters of different aECM/PLGA microspheres, a: PLGA, b: 1aECM/PLGA, c: 5aECM/PLGA, d: 10aECM/PLGA, e: 25aECM/PLGA, f: 50aECM/PLGA, g: 25Col/PLGA). N=20.

**Figure S2.** The viscosity of aECM/PLGA suspension in different ratios. a: PLGA, b: 1aECM/PLGA, c: 5aECM/PLGA, d: 10aECM/PLGA, e: 25aECM/PLGA, f: 50aECM/PLGA, g: 25Col/PLGA).
